# Case Report: Contiguous Xq22.3 Deletion Associated with ATS-ID Syndrome: From Genotype to Further Delineation of the Phenotype

**DOI:** 10.3389/fgene.2021.750110

**Published:** 2021-10-29

**Authors:** Jan Smetana, Vladimira Vallova, Marketa Wayhelova, Eva Hladilkova, Hana Filkova, Vera Horinova, Petr Broz, Aneta Mikulasova, Renata Gaillyova, Petr Kuglík

**Affiliations:** ^1^ Department of Genetics and Molecular Biology, Institute of Experimental Biology, Faculty of Science, Masaryk University, Brno, Czech; ^2^ Department of Medical Genetics and Genomics, University Hospital Brno, Brno, Czech; ^3^ Genetic Ambulance and Counseling, Jihlava, Czech; ^4^ Department of Biology and Medical Genetics, 2nd Faculty of Medicine, Charles University Prague and Faculty Hospital Motol, Prague, Czech; ^5^ Biosciences Institute, Newcastle University, Newcastle upon Tyne, United Kingdom

**Keywords:** ATS-MR syndrome, trio-based whole exome sequencing, genotype-phenotype analysis, neurodevelopmental disorders, Xq22.3q23 deletions

## Abstract

Alport syndrome with intellectual disability (ATS-ID, AMME complex; OMIM #300194) is an X-linked contiguous gene deletion syndrome associated with an Xq22.3 locus mainly characterized by hematuria, renal failure, hearing loss/deafness, neurodevelopmental disorder (NDD), midface retrusion, and elliptocytosis. It is thought that ATS-ID is caused by the loss of function of *COL4A5* (*ATS*) and FACL4 (ACSL4) genes through the interstitial (micro)deletion of chromosomal band Xq22.3. We report detailed phenotypic description and results from genome-wide screening of a Czech family with diagnosis ATS-ID (proband, maternal uncle, and two female carriers). Female carriers showed mild clinical features of microscopic hematuria only, while affected males displayed several novel clinical features associated with ATS-ID. Utilization of whole-exome sequencing discovered the presence of approximately 3 Mb of deletion in the Xq23 area, which affected 19 genes from *TSC22D3* to *CHRDL1.* We compared the clinical phenotype with previously reported three ATS-ID families worldwide and correlated their clinical manifestations with the incidence of genes in both telomeric and centromeric regions of the deleted chromosomal area. In addition to previously described phenotypes associated with aberrations in *AMMECR1* and *FACL4*, we identified two genes, members of tripartite motif family *MID2* and subunit of the proteasome PA700/19S complex (*PSMD10*), respectively, as prime candidate genes responsible for additional clinical features observed in our patients with ATS-ID. Overall, our findings further improve the knowledge about the clinical impact of Xq23 deletions and bring novel information about phenotype/genotype association of this chromosomal aberration.

## Introduction

Alport syndrome with intellectual disability (ATS-ID, AMME complex; OMIM #300194) is classified as an X-linked contiguous gene deletion syndrome associated with an Xq22.3 locus. This syndrome is characterized by hematuria, progressive renal failure, progressive sensorineural hearing loss, ocular changes, intellectual disability (ID), midface hypoplasia, and elliptocytosis (Nozu et al., 2017). Genetic basis of the disease is traditionally associated with incidence of mutation and intragenic deletion of *COL4A5* gene (Lee et al., 2019). Several other studies showed that Xq22.3 locus contains more genes associated with ID and identified two additional genes *AMMECR1* and *FACL4* as important genetic markers of the disease (Lane et al., 1994; Jonsson et al., 1998; Meloni et al., 2002b; Bhat et al., 2006; Rodriguez et al., 2010; Gazou et al., 2013). In addition, *FACL4* (*ACSL4*) was identified as the candidate gene for nonspecific ID for ATS-ID patients (Meloni et al., 2002a).

The utilization of whole-exome sequencing (WES) approach was recently used for the detection of pathological genetic variants of various clinical syndromes and diseases (Zheng et al., 2018; Dai et al., 2019; Han et al., 2020; Zhang et al., 2020). In case of ATS-ID, exome sequencing analyses provided data connecting missense and nonsense pathogenic variants in *AMMECR1* with occurrence of elliptocytosis, cardiac and bone defects, ID, and midface hypoplasia (Andreoletti et al., 2017; Basel-Vanagaite et al., 2017; Moyses-Oliveira et al., 2018). Recently, Poreau et al*.* described unrelated patients with 70- and 146-kbp microdeletions in Xq22.3 affecting *TMEM164* and *AMMECR1*, providing novel evidence for involvement of *AMMECR1* in the ATS-ID phenotype (Poreau et al., 2019).

In this study, we report the clinical and genome-wide characterization of a Czech family with ATS-ID (proband, maternal uncle, and two female carriers). While karyotype assessed by conventional G-banding was normal in all four patients, oligonucleotide array-based comparative genome hybridization (array-CGH) and whole-exome sequencing in this family revealed a 3-Mb deletion including *TSC22D3* at the proximal breakpoint and *CHRDL1* at the distal breakpoint site. We compared the extent of the deleted region with previously published studies and performed a genotype/phenotype correlation study to further clarify the other associated clinical features within this contiguous deletion syndrome. In addition, based on our genome-wide approach, we speculate on potential candidate genes involved in the deleted chromosomal region.

## Case Description

**FIGURE 1:**
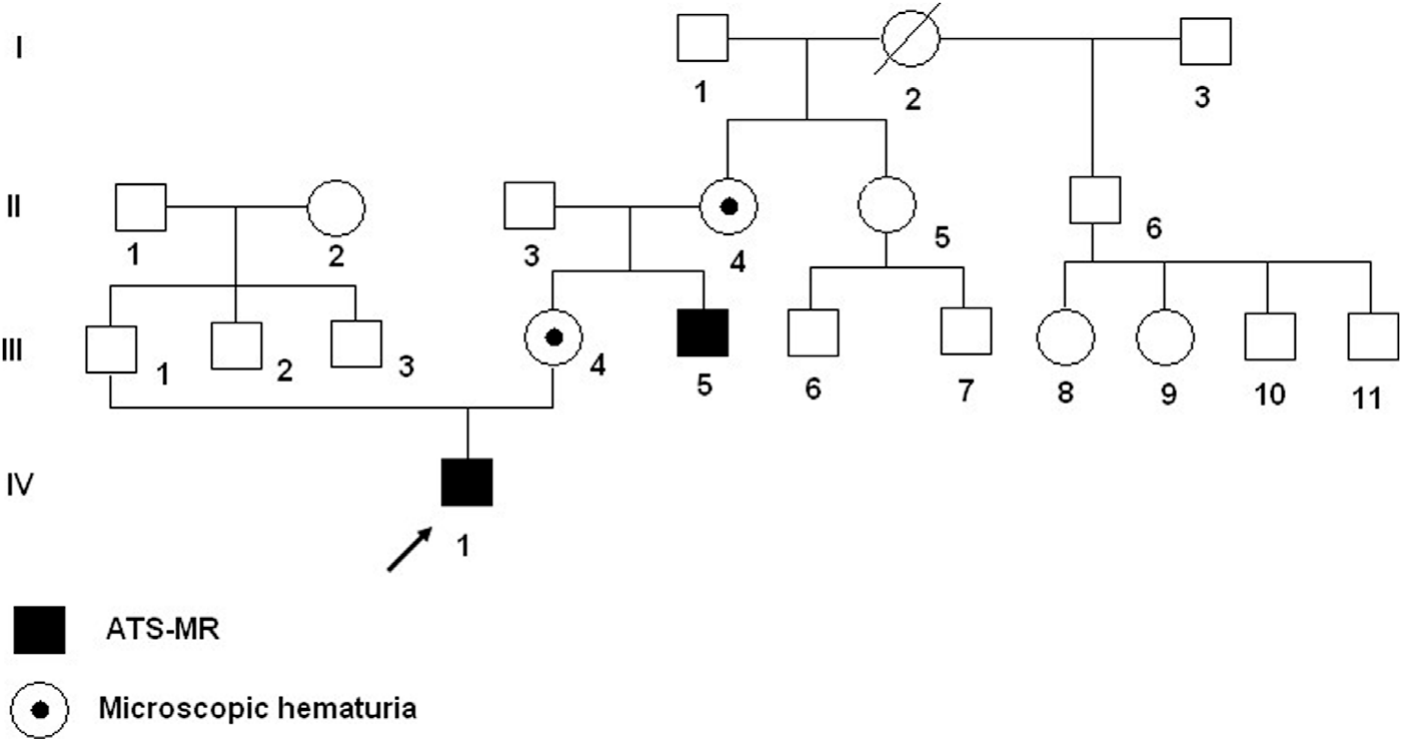
Pedigree of the Czech family with ATS-ID syndrome. Proband (IV-1) and his maternal uncle (III-5) have hemizygous deletion in Xq22.3; the mother (III-4) and grandmother (II-4) are heterozygous for deletion Xq22.3.

A Caucasian Czech family with affected proband and maternal uncle (Figure 1: IV-1 and III-5) with ATS-ID syndrome and carrier mother and maternal grandmother (Figure 1: III-4 and II-4) were referred by the pediatrician to genetic counseling and diagnosis because of suspected neurodevelopmental disorder (NDD).

### Proband

**FIGURE 2:**
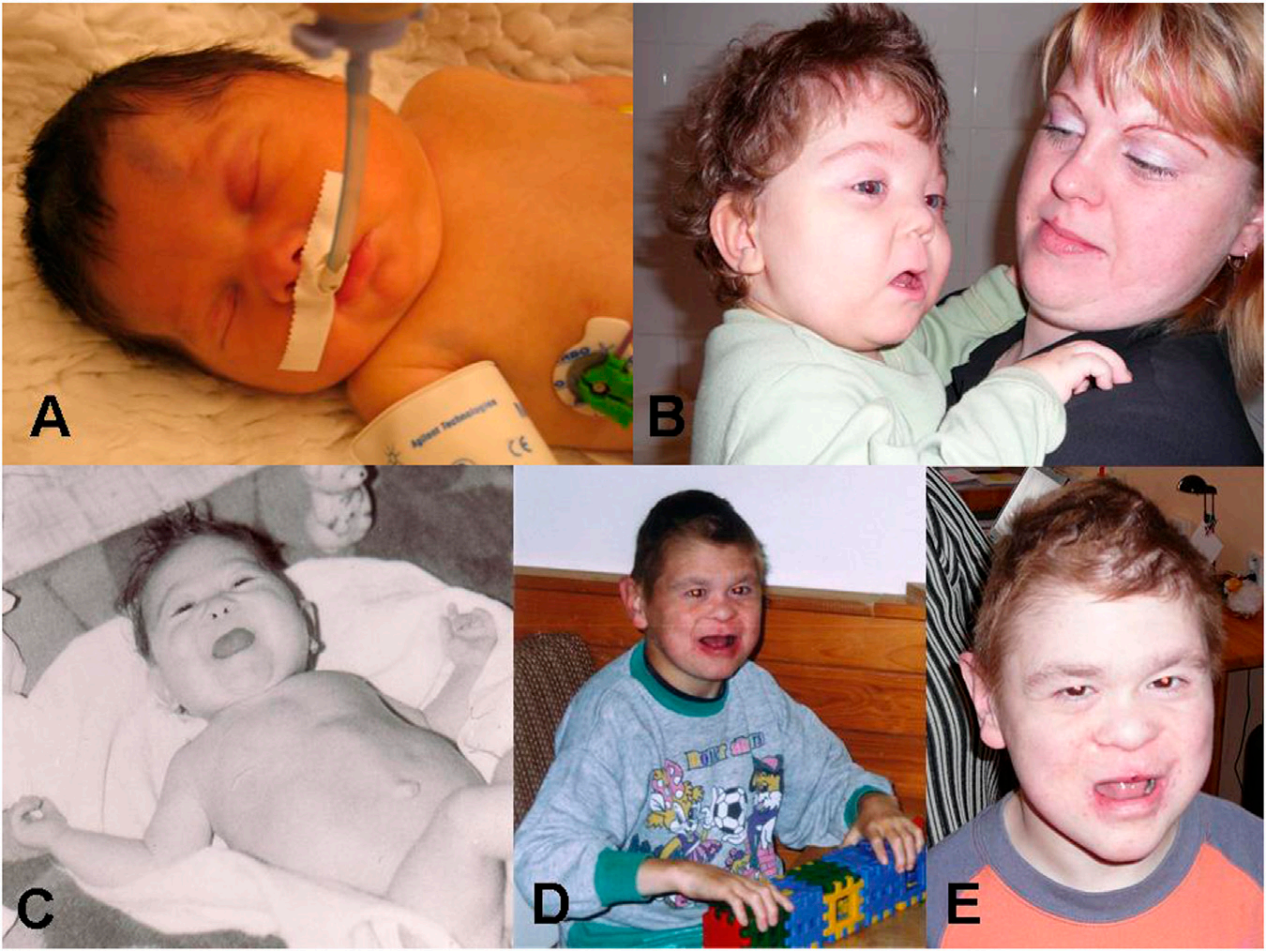
Proband: **(A)** Short after birth. **(B)** At 2 years of age with mother. Maternal uncle: **(C)** At 10 months of age. **(D)** At 17 years of age. **(E)** At 20 years of age.

The proband (Figure 1: IV-1) was born at 37 weeks of gestation with birth weight of 3,200 g and length of 48 cm. He was conceived as the first child from uncomplicated gravidity to a 24-year-old mother with a 30-year-old father, both healthy and nonconsanguineous. Shortly after birth, intubation was needed because of respiration failure. The infant was born with sepsis, hypotonia, neonatal hepatitis of undefined etiology, duplicated left renal pelvis, hyperechogenic kidneys, diffuse axonal injury, pulmonary hypertension, valvular insufficiency, aortopulmonary collaterals, micropenis, and cryptorchidism. Dysmorphic features included brachycephaly and macrocephaly, midface retrusion, hypertelorism, antimongoloid eye slants, epicanthus, synophrys, anteverted nares, dysplastic low-set ears, downturned corners of mouth, and palmar crease on his hands (Figures 2A,B).

Examination at the age of 13 years confirmed most dysmorphic features (epicanthus, hypertelorism, synophrys, microcephaly, dysplastic low-set ears, downturned corners of mouth, palmar crease on both hands, widely spaced teeth, and caries) and showed severe NDD. There is no speech at all (his own few words and crooning); he is better receptive than expressive, and he is able to do simple tasks, but must be fed, spoon-food only. He is not able to keep cleanliness requirements and has fits of laughter. Also, attention deficit, instability, and mild aggressiveness were observed. He started to walk at 3 years; his walk is tentative with ataxic gait. At 13 years, the proband is approximately 140 cm in height with beginning puberty (pubic hair). Testes are *in situ* with normal male genitalia.

The proband has partial deafness (40%). He underwent cataract surgery (both lens implantation at 8 and 9 years of age). He is monitored at the cardiology (he underwent closing of arterial duct), neurology, and nephrology with no problems of the kidneys so far.

### Maternal uncle

The maternal uncle (III-5) was born at 39 weeks of gestation (weight = 2,900 g, length = 49 cm) from second uncomplicated gravidity to a 25-year-old mother and a 28-year-old father, both healthy and nonconsanguineous. He was born with glucosuria, hyperbilirubinemia, pulmonary hypertension, and dysmorphic features, including flat midface, hypertelorism, antimongoloid slants, deep-set eyes, synophrys, anteverted nares, hypoplastic nasal bones, downturned corners of mouth, sparse hair, palmar crease, cutaneous syndactyly of two to three fingers, micropenis, cryptorchidism, cutis marmorata, and hypertrichosis (Figures 2C–E). In a later age, hepatopathy, decreased function of left kidney, hereditary glomerulopathy, short stature (150 cm in adultness), and severe developmental delay were described. He did not speak at all, and his walk was tentative. There also was attention deficit, instability, and mild aggressiveness, but no fits of laughter were noticed. He died at the age of 27 because of kidney failure.

The mother and maternal grandmother were heterozygous with manifested microscopic hematuria, but both were of normal intelligence.

#### Whole-Exome Sequencing

**FIGURE 3:**
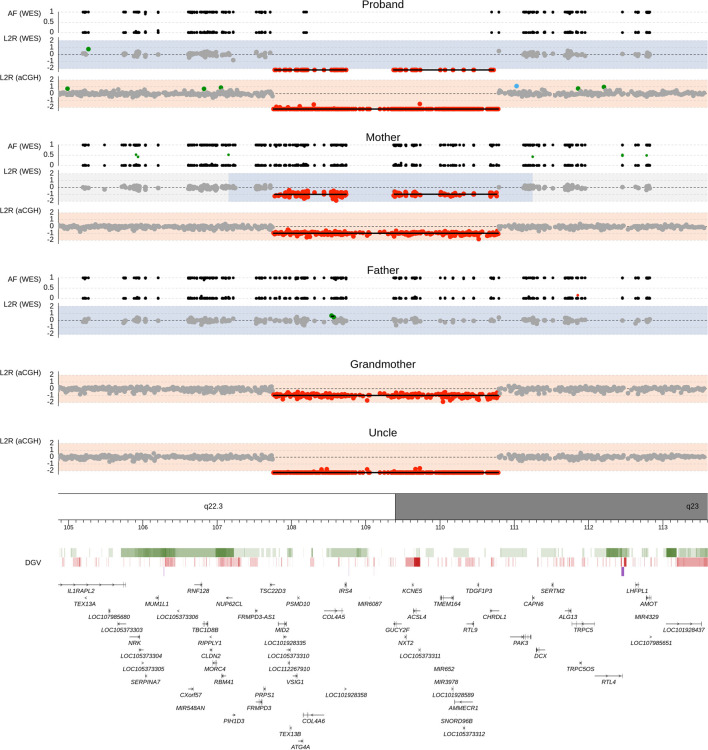
Chromosomal deletion del(X) (q22.3q23) detected by whole-exome sequencing and array-comparative genome hybridization (CGH). Upper panel shows genomic profiles of the proband, father, and mother. Log2 ratio (L2R) track represents the copy-number profile indicating a maternally inherited hemizygous deletion in the male proband. Gray dots mark sequencing targets without any copy number abnormality. Red and green dots indicate the presence of losses and gains, respectively. Areas with light blue background show loss of heterozygosity regions predicted by zygosity status as shown in the allele frequency (AF) plot. In the AF track, black dots represent SNPs with a single allele detected in the sequencing reads, and green and red dots highlight SNPs with two alleles found in approximately 1:1 and another ratio, respectively. Lower panel contains the Database of Genomic Variants (DGV) and RefSeq gene annotation database. Color intensity within the DGV track corresponds to the level of population variability, green for losses, red for gains, and purple for complex regions.

The proband and his parents were then examined by trio-based WES approach using commercially available Human Core Exome kit (Twist Bioscience) on Illumina NovaSeq 6000. Using our WES pipeline, we acquired a total of 226,580 genetic variants, of which we identified overall 151 filtered SNPs and indel variants, and all were classified as benign/likely begin or uncertain significance (see List 1 of Supplementary Table S1). In addition, two different segmentation algorithms for copy number variation (CNV) evaluation identified a Xq22.3q23 deletion of size approximately 2.91 Mb in the proband and his asymptomatic mother (Figure 3).

In order to confirm the WES results, we performed array-CGH in four family members (proband, mother, maternal grandmother, maternal uncle). The results showed loss of genetic material in the Xq22.3 locus [arr (hg19) Xq22.3q23 (107001860_110026871)x0] of size over 3.03 Mb, which affected the area comprised of 19 genes from *TSC22D3* to *CHRDL1.* Data from WES and array-CGH can be found in The European Genome-phenome Archive (EGA) (Lappalainen et al., 2015) under datasets IDs EGAD00001007740 and EGAD00001007743, respectively.

#### Cytogenetic and qPCR Analysis

Cultured metaphase chromosomes from peripheral blood lymphocytes were used for examination by standard G-banding karyotyping with normal results in the proband (IV-1) and his parents (III-1, III-4), maternal uncle (III-5), and grandmother (II-4).

**FIGURE 4:**
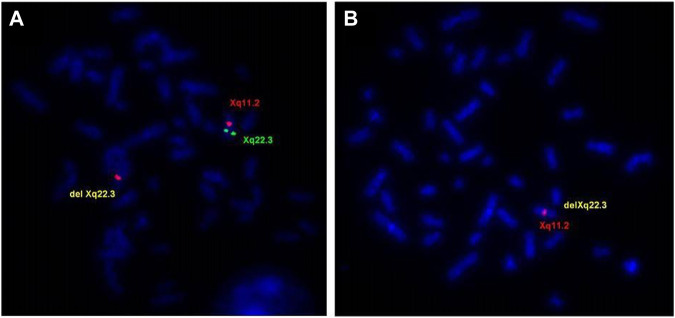
FISH evaluations of the delXq22.3 region. **(A)** Metaphase chromosomes from the mother (III-4) showed FISH signal for the Xq22.3 region obtained using probe RP1-31B8 (green) on only one X chromosome. Probe for X chromosome centromeric region (RP13-34C21—red) showed FISH signals in both chromosomes. **(B)** Metaphase chromosomes from the proband (IV-I) showed no FISH signal for the Xq22.3 region and only one signal for the X chromosome centromeric region.

FISH analysis using a probe RP1-31B8 (108,414,862–108,517,413), which spans the proximal region of *GUCY2F* gene, confirmed the deletion in the proband and in the affected maternal uncle (IIII-5) (Figure 4B). The mother (III-4) and grandmother (II-4) were shown to be the carriers of the Xq22.3 deletion (Figure 4A), and the sister of the grandmother (II-5) was negative.

Deletion breakpoints were analyzed by qPCR together with a combination of WES and array-CGH results. Those breakpoints were detected between exon 1 and exon 2 of the *TSC22D3* gene (NM_198057.3) for the centromeric and between exon 2 and exon 3 of the *CHRDL1* gene (NM_001367204.1) for the telomeric one (see Supplementary Methods).

## Discussion

Alport syndrome with intellectual disability (ATS-ID, AMME complex; OMIM #300194) is an X-linked syndrome associated with the Xq22.3 loci. ATS-ID is a rare hereditary disorder with prevalence of about 1 in 50,000 live births (Kashtan et al., 2013). We reported the first Czech family affected with this congenital syndrome.

### Comparison With Previously Reported ATS-ID Cases

A comparison of the deleted Xq22.3 area and clinical features in the reported family and three previously reported families with ATS-ID syndrome are shown in List 2 of Supplementary Table S1.

The 2.91-Mb region of loss of genetic material in our reported family is so far the largest of all described deletions in this region; it extended more telomerically and spans additional genes in comparison with the other previously described Xq22.3 deletions including *MID2*, *VSIG1*, *TEX13B*, *ATG4A*, *PSMD10*, *TDGF3*, *CHRDL1*. These findings could clarify the minor clinical differences observed in affected members of the Czech family and affected members of previously published families and bring novel information about the potential of candidate genes involved in the deleted segment. The clinical symptoms observed in our family, and not previously described in other families, included neonatal hepatitis, duplicated renal pelvis, aortopulmonary collaterals, palmar crease, growth retardation, micropenis, and cryptorchidism. We speculate that one or more of the genes found in our additional deleted region may be the causal gene for the symptoms, which were not described in previous families.

At present, the most interesting candidate gene is *MID2*, which encodes the protein midline 2 isoform 2, a member of the tripartite motif (TRIM) family. *MID2* is a member of E3 ubiquitin ligase subclass with a role in the microtubule stabilization in the cytoplasm (Short and Cox, 2006). *MID2* is closely related to the *MID1* with sequence similarity; *MID1* and *MID2* also share the same subcellular localization, but they have a different expression pattern during development (Buchner et al., 1999). To the best of our knowledge, there are no conclusive data connecting deregulation of *MID1* and *MID2* genes to the ATS-ID phenotype. However, *MID1* is the gene responsible for the Xp22-linked form of Opitz syndrome (OMIM 145410). This syndrome is a genetically and phenotypically complex disorder. It is defined by characteristic facial anomalies (hypertelorism, variably labiopalatine, laryngotracheo-esophageal clefting), structural heart defects, and anal and genital anomalies (Quaderi et al., 1997). Opitz syndrome also shares many anomalies with another X-linked disorder, FG syndrome (OMIM #305450), especially of midline structures. FG syndrome is characterized by multiple congenital anomalies (cleft palate, heart defects) and a broad range of central nervous system (CNS) dysfunctions (such as hypotonia, delay in motor development, speech delay) (Graham and Schwartz, 2013).

The clinical similarity between Opitz and FG syndromes, together with the sequencing homology between *MID1* and *MID2*, is probably the main reasons for considering *MID2* as a positional and functional candidate gene for FG syndrome (Jehee et al., 2005).

Both described syndromes are associated with congenital heart malformation, in case of Opitz syndrome additionally with genital anomalies. We speculate that *MID2* may play a role in the development and could be responsible for the heart defect (aortopulmonary collaterals) or genital anomalies (micropenis, cryptorchidism) or kidney defect (duplicated renal pelvis) and facial dysmorphism, including hypertelorism, in our family. This hypothesis must be verified by additional detailed analysis confirming the exact role of the *MID2* gene.

Until now, there is little information about the other genes, which were deleted in our case. The *VSIG1* gene is expressed in the normal testis and stomach, as well as in esophageal, ovarian, and gastric cancers (Oidovsambuu et al., 2011; Inoue et al., 2017), the *TEX13B* that is expressed in the testis during spermatogonia (Wang et al., 2001). *PSMD10* encodes 26S proteasome non-ATPase regulatory subunit (Gankyrin), which is essential for ubiquitin-dependent protein degradation (Hori et al., 1998). In addition, overexpression of *PSMD10* has been implicated in the development of many cancer types (Chattopadhyay et al., 2016). With the pivotal role in the ubiquitin–proteasome system (UPS), accumulation of this protein may be associated with the pathogenesis and phenotypic expression in several malignancies (Ermolaeva et al., 2015), cardiovascular, autoimmune, and neurodegenerative diseases including Alzheimer’s disease, muscular dystrophies, and rare forms of neurodegenerative diseases associated with the development of dementia (Checler et al., 2000; Mathews and Moore, 2003; Mayer, 2003). While there are no conclusive data about degradation of synaptic proteins due to CNVs affecting *PSMD10* (Piton et al., 2011), we hypothesize that deregulation of *PSMD10* expression through loss of the genetic material in the locus could contribute to severe MR and developmental delay in our proband and maternal uncle. The effects of CNVs in *ATG4A*, *TDGF3*, and in the pathogenesis of MR have not been yet described.

A detailed comparison showed that patients in our study were similar in terms of clinical presentation. They shared similar major clinical characteristics with the previously reported ATS-MR phenotype. The difference in the phenotypes could be explained most probably due to deregulated expression of *MID2* and *PSMD10*. While evidence obtained from the literature indicate craniofacial abnormalities corresponding to midface retrusion seen in affected males, further functional, e.g., expression analysis of *MID2* and *PSMD10* need to be performed in order to verify the effect of deregulated expression on the phenotype. In addition, our detailed description of phenotype of the 20-year-old uncle might also be useful for future prediction of the development of the symptoms in other patients. Finally, the identification of CNVs in the Xq22.3 locus could be essential for genetic counseling strategies for other younger patients.

## Data Availability

The datasets presented in this study can be found in online repositories. The names of the repository/repositories and accession number(s) can be found in the article/Supplementary Material.
