# Supplementary material for: Case Report: Contiguous Xq22.3 Deletion Associated with ATS-ID Syndrome: From Genotype to Further Delineation of the Phenotype
Source: Front Genet. 2021 Oct 29;12:750110. doi: 10.3389/fgene.2021.750110 (PMC8585740; doi:10.3389/fgene.2021.750110)
Supplement: Supplementary file 2 [file DataSheet1.docx]

# **Biological material and DNA extraction**

The proband was diagnosed at the Department of Medical Genetics (University Hospital Brno). All biological samples were obtained only after the patients signed the informed consent approved by the Ethical committee of University Hospital Brno, Czech Republic

Genomic DNA samples were obtained from 1 ml peripheral blood in EDTA, according to the standard DNA isolation process using the MagNaPure system (Roche Diagnostics, Basel, Switzerland). Quality and quantity were checked using a DeNovix DS-11 Spectrophotometer (DeNovix Inc., Wilmington, DE, USA) and Qubit^®^ 2.0 (Thermo Fisher Scientific, Inc., Waltham, MA, USA).

# **Methods**

## *Whole-exome sequencing (WES)*

Approximately 200 ng of high-quality of genomic DNA samples of proband and his parents were used for library preparation. DNA libraries were prepared using the Human Core Exome Kit according to manufacturer’s recommendations (Twist Bioscience, San Francisco, CA, USA) and then sequenced on Illumina NovaSeq 6000 (Illumina, Inc., san Diego, CA, USA). All steps were performed as a commercially available service (Institute of Applied Biotechnologies, Czech Republic).

### WES data processing and variant analysis

Raw sequencing data were processed to obtain both sequence (SNVs, indels) and structural variants. Quality control was performed FastQC v. 0.11.8 following loq quality reads trimming and adapter contamination by the fastp 0.20.0. The remaining reads were aligned to reference human genome hg38 / GRCh38 primary assembly by BWA 0.7.17-r1188 with default parameters following by marking PCR and duplicates reads. Quality control steps and coverage control was performed in-house software Genovesa. Variant calling was done using VarScan v2.4.4 (with parameters --min-coverage 30, --min-var-freq 0.1).

Copy-number variants (CNV, CNVs) analysis is based on read of depth algorithms. Filtering of low-quality reads and adapter contamination were performed by the fastp 0.20.0. The remaining reads were aligned to reference human genome hg38 / GRCh38 primary assembly by BWA 0.7.17-r1188 with default parameters following by marking PCR and duplicates reads. Then we used the 35-mer mappability score form UCSC genome browser across each exon and then we exclude any exon with mappability score lower than 0.75. R software version 3.6.0 was used to and calculate and normalized coverage inside region of exons targets (defined by BED file). Algorithm need at least 6 sample to create read depth coverage base line and then compares each sample to each. The ration of expected reads to real number of reads is calculated and then can be estimated gain or loss in specific locus defined by target.

Applying an alternative segmentation algorithm, CNVs were detected using custom pipeline based on sequencing depth in whole-exome sequencing data (Mikulasova *et al.*, in preparation). GATK tools v4.1.4.0 (Broad Institute, Cambridge, MA, USA) were used to process bam files and data denoising. Allele-frequency data was obtained from gnomAD v2.1 (Broad Institute). CNV and loss of heterozygosity (LOH) segments were called using the in-house algorithm and filtered by parameters as follows: CNV – minimum of 50bp and 2 intervals, less than -0.5 Log2 Ratio (L2R) for losses and more than 0.3 L2R for gains; LOH – minimum of 4Mb and 10,000 intervals. Variant Effect Predictor v98 (Ensembl, The European Bioinformatics Institute, Hinxton, UK) was used for gene annotation. Unaffected unrelated sex-matching individuals (19 males and 19 females) were used as controls for data denoising. All variants were manually inspected to determine data quality, inheritance, population variability using Database of Genomic Variants (The Centre for Applied Genomics, Toronto, Canada), and presence of CNVs in controls.

### Variant filtering and data analysis

Only SNVs or indels with minimal quality of coverage ≥30X, base quality ≥10, mapping quality ≥7 and with alternative allele frequency ≥20% per sample, p-value (Fisher exact test) 0.05 were included for further variant filtering. For CNVs we considered only those detected by both segmentation algorithms for further filtering, classification and interpretation.

Using trio-based analysis, we primarily focused on exonic SNVs or indels with the putative impact “high” and “moderate” and applied filtering for rare variants (gnomad non-Finnish European allele frequency ≤5%), For recurrent variants we considered pathogenic and likely pathogenic clinical significance based on ClinVar [1]. Novel variants in OMIM “morbid” genes were classified using VarSome and only pathogenic or likely pathogenic variants with clinical relevance were then reported [2].

The CNVs reported by both algorithms were then filtered for the content of OMIM “morbid” genes with clinical relevance and thus were classified as pathogenic or likely pathogenic. The remaining CNVs lacking clinically relevant genes were classified according to general guidelines [3]. The clinical interpretation was performed using relevant databases, as ClinVar, OMIM [4], DECIPHER [5] etc.

### Manual curation

Clinically relevant CNVs were manually checked using Integrative Genomic Viewer with parallel visualization of bed files obtained by both segmentation algorithms for the whole family trio. The presence of CNV was confirmed by two independent CNV pipelines and manually curated to get minimal CNV region. Array-CGH and real-time PCR using specific primer pairs were performed to validate reportable, clinically-relevant CNV.

## *Microarray analysis*

Whole-genomic screening of submicroscopic unbalanced chromosomal rearrangements by array-CGH using oligonucleotide DNA microarray platform SurePrint G3 Human CGH Microarray, 4X180K (AMADID #022060; Agilent Technologies, Santa Clara, CA, USA) was performed to validate CNVs detected by WES. Microarray data were obtained and visualized by Agilent Cytogenomics, 4.0.3. CNVs were detected using the ADM-2 algorithm with the filter ≥ consecutive probes in genomic region, minimal size of 100 kb in region and minimal absolute log_2_ ratio 0.25. All genomic positions were assessed on the human reference genome GRCh37/hg19. Genomic positions for clinically relevant CNVs obtained by array-CGH were then converted to GRCh38/hg38 for curation of WES and array-CGH data. The CNVs were classified as recommended by general guidelines [3].

## *Real-time PCR*

Relative qPCR was performed with six pairs of DNA primers which were designed to prime the DNA sequence in the close proximity of the first deleted array-CGH probe (*TSC22D3* gene) and into exons 2, 3 and 4 of the *CHRDL1* gene. One primer pair targeting the *ERH* gene was used as an endogenous control, the Human Reference DNA Male (Agilent Technologies) was used as a reference sample. The reactions were performed using Power SYBR^TM^ Green Master Mix and run in duplicates, following the manufacturer’s recommendations (ThermoFisher Scientific). C_T_ values were subtracted for the ERH gene and each tested DNA region, for which was evaluated to derive R-values. An R-value ~ 0 for DNA loss and ~ 1.0 for normal relative DNA amount of the targeted DNA regions relatively to the ERH gene were set as cut-offs. The DNA primers were synthesized using a commercially available service (Integrated DNA Technologies, Coralville, Iowa, USA; (Table 1)

Table 1. Primer designs for qPCR

| Primers for CNV verification using real-time PCR (qPCR) | | | |  |  |
| --- | --- | --- | --- | --- | --- |
| primer | forward primer (5' - 3') | reverse primer (5' - 3') | PCR product range (GRCh38/hg38) | | genomic location |
| TSC22D3_1 | AGAATCTGTTCCCCGGTGTT | ACATCTTGCCCACAGAATGC | chrX:107,756,984-107,757,071 | | TSC22D3, intron 1 |
| TSC22D3_2 | CACTTTGCCCAGCAGTGTAG | AACCAGAGAGTGCCTTTCCA | chrX:107,758,226-107,758,344 | | TSC22D3, intron 1 |
| TSC22D3_3 | CATGGGATCACTGGGAGACA | TGGGCTTGGAACGGATTAGT | chrX:107,758,766-107,758,869 | | TSC22D3, intron 1 |
| CHRDL1_1 | TGGAGAACCACAGCTAGGAAA | AGCCGAGTCAGATGTCCAAA | chrX:110,759,619-110,759,736 | | CHRDL1, exon 4 |
| CHRDL1_2 | GCAGTTCACGCAGTAAACCA | AGGCATATTCTCCATCTCCTTCT | chrX:110,762,707-110,762,836 | | CHRDL1, exon 3 |
| CHRDL1_3 | ACGTTTTACTTGCTCTGTTTTGC | TGAAGGTTCTGTCACCTTTTGC | | chrX:110,792,086-110,792,213 | CHRDL1, exon 2 |

## G-banded karyotype

Metaphase slides from cultivated peripheral blood lymphocytes were prepared using standard methanol:acetic acid fixation (3:1), treated by trypsin (Sigma-Aldrich, Prague, Czech Republic) and stained by Giemsa solution to obtain chromosomal G-banding karyotype in resolution of 550 bands.

## FISH (fluorescence *in situ* hybridization)

FISH analyses were performed applying the RP1-31B8 SG (Xq22.3) and RP13-34C21 SR (Xq11.2) Probes (BlueGnome, Cambridge, UK) following the manufacturer’s instructions on cultured peripheral blood lymphocytes (chromosomal metaphase slides).

## Data availability

All sequencing and array-data were successfully submitted to The European Genome-phenome Archive (EGA) [6]. Files for WES and array-CGH can be found under datasets IDs EGAD00001007736 and EGAD00001007743, respectively.

The other supporting data from validation analysis by G-banding, FISH or qPCR are available from the corresponding author on the reasonable request.

# References

1. Landrum, M.J., et al., *ClinVar: public archive of relationships among sequence variation and human phenotype.* Nucleic Acids Research, 2013. **42**(D1): p. D980-D985.

2. Kopanos, C., et al., *VarSome: the human genomic variant search engine.* Bioinformatics, 2019. **35**(11): p. 1978-1980.

3. Silva, M., et al., *European guidelines for constitutional cytogenomic analysis.* Eur J Hum Genet, 2019. **27**(1): p. 1-16.

4. Hamosh, A., et al., *Online Mendelian Inheritance in Man (OMIM), a knowledgebase of human genes and genetic disorders.* Nucleic Acids Res, 2005. **33**(Database issue): p. D514-7.

5. Firth, H.V., et al., *DECIPHER: Database of Chromosomal Imbalance and Phenotype in Humans Using Ensembl Resources.* Am J Hum Genet, 2009. **84**(4): p. 524-33.

6. Lappalainen, I., et al., *The European Genome-phenome Archive of human data consented for biomedical research.* Nature Genetics, 2015. **47**(7): p. 692-695.
